# Differential physiological changes following internet exposure in higher and lower problematic internet users

**DOI:** 10.1371/journal.pone.0178480

**Published:** 2017-05-25

**Authors:** Phil Reed, Michela Romano, Federica Re, Alessandra Roaro, Lisa A. Osborne, Caterina Viganò, Roberto Truzoli

**Affiliations:** 1 Swansea University, Swansea, United Kingdom; 2 Università degli Studi di Milano, Milan, Italy; 3 Abertawe Bro Morgannwg University Health Board, Swansea, United Kingdom; Universita Cattolica del Sacro Cuore Sede di Roma, ITALY

## Abstract

Problematic internet use (PIU) has been suggested as in need of further research with a view to being included as a disorder in future Diagnostic and Statistical Manual (DSM) of the American Psychiatric Association, but lack of knowledge about the impact of internet cessation on physiological function remains a major gap in knowledge and a barrier to PIU classification. One hundred and forty-four participants were assessed for physiological (blood pressure and heart rate) and psychological (mood and state anxiety) function before and after an internet session. Individuals also completed a psychometric examination relating to their usage of the internet, as well as their levels of depression and trait anxiety. Individuals who identified themselves as having PIU displayed increases in heart rate and systolic blood pressure, as well as reduced mood and increased state of anxiety, following cessation of internet session. There were no such changes in individuals with no self-reported PIU. These changes were independent of levels of depression and trait anxiety. These changes after cessation of internet use are similar to those seen in individuals who have ceased using sedative or opiate drugs, and suggest PIU deserves further investigation and serious consideration as a disorder.

## Introduction

Problematic internet use (PIU) is a global issue [1], which is thought to be most pronounced among younger individuals [2,3], but which is recognized as in need of further study in order to define its precise nature [1,4–6]. PIU has been found to be associated with a range of psychological problems, such as depression [7], anxiety [8,9], social isolation [10], and impulsivity [11,12], and these associations have been well reviewed [13–15]. Those scoring highly on measures of PIU also report experiencing psychological withdrawal effects after cessation of contact with the internet [16–18]. These withdrawal effects have been found to include increased negative mood [17], depression [16], and anxiety [19]; in addition, cravings have also been noted for those reporting internet video game addictions [20]. However, the impact of internet cessation is still poorly understood at the physiological level, and this represents a major gap in knowledge.

Although it is not entirely clear how PIU should be classified in terms of a potential disorder, there is mileage in exploring putative physiological withdrawal effects in terms of known impacts of cessation of addictive substances. In terms of substance addiction, physiological measures (e.g., heart rate, blood pressure) are immediately affected by cessation of use of the substance in question. The nature of the effect of cessation on the physiology of the individual is dependent upon the substance. For example, cessation of alcohol [21], cannabis [22], and opiate [23], use leads to an increase in such physiological measures in those who abuse these substances. Whereas, cessation of usage of MDMA [24,25], nicotine [26], and stimulants [25], depresses these physiological systems in individuals addicted to them. It is currently unknown whether internet cessation would have any such effect on those who claim PIU, and, although such physiological effects do not describe withdrawal effects in their entirety [18], without such information the potential classification of PIU as a particular form of disorder is made difficult [4–6].

Given this gap in current knowledge, the present study explored the impact of internet cessation on the physiological functioning of those who reported higher- or lower-PIU. As with the previous explorations of these issues for those impacted by addictive substances [21–23], blood pressure and heart rate were used as the physiological measures; with state anxiety and mood being the psychological variables measured, as these have been noted previously to be impacted by internet cessation [16,17]. These variables were measured immediately prior to and after an internet session, and changes in these variables were noted in order to assess the impact of cessation of exposure to the internet [17,27]. To rule out the influence of psychological variables that are often associated with PIU (i.e. depression [9] and trait anxiety [8]), and which may impact physiological changes, these factors were also assessed for the participants, and included in the analyses as covariates. If those who self-report higher levels of PIU display psychological withdrawal effects akin to those seen previously, then there should be an increase in state anxiety and changes in mood following cessation of the internet session [16–19,27]. Furthermore, if these individuals show physiological withdrawal effects akin to those seen for individuals addicted to substances, there should also be changes in their physiological functioning following cessation of the internet session. However, the nature of the physiological changes produced by the cessation were not predicted, as it is currently unknown how (or if) internet use functions in such a manner for those who self-report PIU.

## Method

### Ethical statement

Ethical approval for this research was obtained from the Department of Psychology Ethics Committee, Swansea University. The participants provided informed consent to participate in this study by signing a consent form following reading the information sheet provided for them, and the Ethics Committee approved this consent procedure.

### Participants

Previous examinations of the changes in psychological state as a result of internet exposure have identified medium-sized effects [12,17,27], as have those investigating the physiological impact of substance cessation [21–23]. Assuming such medium effect-sizes (*d* = .5) between lower- and higher-PIU groups in the current study, for a power of .80, and adopting a significance criterion of *p* < .05, then an *N* of 128 would be necessary to identify differences.

One hundred and fifty-six volunteers were recruited from a student subject pool, and received subject pool credit for their participation. A student sample was though appropriate as younger individuals are thought to be more at risk from PIU [2,3]. Participants responded to advertisements placed on the subject pool website. After they had volunteered, the participants were asked to report whether they had a history of psychiatric problems (depression, anxiety, psychosis, neurodevelopmental or personality disorder) or addictions to substances or to activities, such as gambling (on or offline). Twelve participants reported that they had such a history, and their data were excluded from the study. It was hoped that this exclusion would limit some potential comorbid problems influencing the results. This left 144 participants (81 females; 63 males) in the study, who had a mean age of 21.26 (SD ± 3.08; range = 18–33) years.

The participants’ self-reported ethnicity was: 57% White; 5% Mixed/Multiple Ethnic Groups; 23% Asian; 16% Black/African/Caribbean; and 1% Other Ethnic Group. Of these participants, 82% reported their nationality as British, but all participants were currently resident in Great Britain. The marital status of the sample was: 88% single, 7% married or in a civil partnership; and 3% in other forms of relationship, and 2% divorced.

### Participant’s typical use of the internet

Participants were asked to estimate the number of hours per week that they had spent on the internet over the last two months; this measure is commonly taken in studies of problematic internet use [12,28,29]. Although it has been suggested that ‘non-professional’ usage correlates most strongly problems associated with heavy internet use [28], it was thought that the professional/non-professional distinction may not apply, or be discriminable, to all of the current participants, and, as total internet use, in itself, also is associated with internet-related problems [28,30], this measure was employed.

The mean number of hours per day of internet use reported was 5.17 (± 4.14, range = 1 to 20): 38.2% reported spending under 3 hours/day online; 38.9% reported spending 3–6 hours/day online; 9% reported spending 6–9 hours/day online; and 13.9% reported spending over 9 hours a day on the internet. The mean number of hours per day spent online by females was 4.85 (± 3.81, range = 1–20), and for males this was 5.57 (± 4.55, range = 1–20). An independent group t-test revealed that this difference was not statistically significant, *t*(142) = 1.03, *p* > .30, *d* = .17.

The participants were also asked to indicate whether or not they had regularly visited particular types of internet site during the last two months. The percentage of the sample who indicated that they had visited such sites was: Social Networking (e.g., Facebook, Twitter) = 91.6%; Shopping/Banking = 90.3%; Research = 84.0%; TV and film = 69.4%; News = 68.7%; Dating and Sexual = 56.3%; Content Sharing (e.g., My Space, You Tube postings) = 45.1%; Gambling (including lottery sites) = 34.7%; Gaming = 24.3%; Traditional Blogging (excluding Twitter) = 20.1%; Chat rooms = 13.2%.

### Materials

#### Internet addiction test (IAT [31])

The IAT is a 20-item scale covering the degree to which use of internet disrupts everyday life. The overall score ranges from 20 to 100. The factor structure of the IAT is currently debated [31,32], but a cut-off score of 40 or more for the total IAT score has been taken as representing some level of problematic internet usage [17,32,33]. The internal reliability of the scale has been found to be between .90 and .93 [33], and this was calculated as .92 for the current sample). The scale also has good concurrent validity with other measures of disruptive internet use [29].

#### Positive and negative affect schedule (PANAS [34])

The PANAS is a 20-item questionnaire designed to measure participants’ positive and negative moods. The total scores can range from 10–50. The internal reliabilities of both the positive and negative scales have been reported as above .90 [34], but these were calculated as .82 for the current sample. The scale also correlates well with other measures of mood, such as the Hospital Anxiety and Depression Scales [35].

#### Spielberger state-trait anxiety inventory (STAI [36])

The STAI rates the affective, cognitive, and physiological manifestations of anxiety in terms of long-standing patterns (trait anxiety) and current anxiety (state). The total score for each scale ranges from 20 to 80. The internal reliability of the scales has been noted to be greater than .90 [36], but this was calculated as .84 for the state and .87 for the trait measure for the current sample). There is strong evidence regarding its validity [36].

#### Beck’s depression inventory (BDI [37])

The BDI is a 21-item questionnaire that assesses the clinical symptoms of depression through asking about feelings over the past week. The score ranges from 0 to 63. The internal reliability of the scale is 0.93 [37] (this was calculated as .94 for the current sample). Reviews have noted that the scale has excellent validity [38].

#### Physiological measures

A heart rate monitor was placed on the index finger of the participants’ non-dominant hand, which they wore for the duration of the study. Heart rate was measured continuously throughout this period, and recorded on a PC. Blood pressure was measured by an automatic electronic monitor.

### Procedure

The participants initially experienced a 2-hour individual teaching session, unrelated to issues concerned with the internet, which took place in the University. Participants were explicitly told to switch off their mobile devices, and they had no access to a PC in the room. The 2-hour period free of the internet prior to the study was chosen as it is similar in length to those periods chosen for studies of cessation of substance use [39], and it corresponded to the longest period of individual teaching scheduled in the timetable (which was organizationally fortuitous and would not make the session seem out of the ordinary for the students).

All subsequent testing was completed individually, in small experimental cubicles. Participants were told that this was an assessment of personality and physiology. They were connected to the physiological equipment, and allowed 15min to acclimatize, while they completed the BDI, STAI-state and trait, and PANAS, measures. During the 2min period, immediately prior to their internet session, their blood pressure was taken twice (the second reading was employed), and their heart rate was collected.

They then had a 15min period of internet exposure in which they could browse whatever sites they wished. The period of exposure to the internet was short to be in line with previous studies of the impact of internet use on psychological and behavioral function [12,17], but also because current usage of the internet is largely through mobile devices, on which people spend many but short sessions, rather than the previously longer sessions at a PC [19,27]. The number and nature of the websites visited was recorded for each participant. Participants were not informed that this information would be collected, as this may have compromised their typical usage of the internet during this session.

In the 2min period immediately following the internet session, blood pressure of the participants was again taken and their heart rate was calculated. Participants then completed the PANAS, STAI-state, and finally the IAT (which was presented after the rest of the experiment was completed, so as not to influence the participants’ other responses as a consequence of seeing this questionnaire), as well as answering questions regarding their use of the internet (estimating how many hours per day, and stating which sites they had typically used, over the past two months).

## Results

The mean internet addiction (IAT) score for the sample was 40.04 (± 13.38; range = 10–73; 46.5% scoring above the cut-off for mild or worse, IAT = 40^22,24^). The mean IAT score for males was 41.84 (± 14.36; range = 10–73; 49% scoring above cut-off); and the mean IAT for females was 38.59 (± 12.48; range = 17–73; 42% scoring above cut-off). The difference in IAT scores between the genders was not statistically significant, *t*(145) = 1.42, *p* = .149, *d* = .24. There was a significant positive correlation between time spent online and the IAT score, *r* = .188, *p* < .05.

Table 1 shows Pearson correlations between the various measures at baseline. Inspection of these correlations shows that the relationships between the variables data were consistent with previous explorations of these measures: PIU scores (measured by the IAT) was associated with both trait and state anxiety, as well as with increased heart rate.

**Table 1:** Mean (standard deviations) for baseline measures and their Pearson correlation coefficients (underlined = *p* < .01; bold and underlined = *p* < .001).

	Mean (SD)	Dep	TA	SA	PM	NM	SP	DP	HR
Internet Addiction (IAT)	40.04 (13.38)	.188	**.303**	**.353**	.004	.127	.149	.105	**.260**
Depression (Dep)	7.00 (4.92)		**.512**	**.292**	.024	**.488**	**.387**	**.315**	.186
Trait Anxiety (TA)	34.58 (12.39)			**.720**	-.171	**.311**	**.457**	**.376**	-.029
State Anxiety (SA)	33.31 (11.20)				-.273	.254	.268	.210	-.138
Positive Mood (PM)	29.19 (6.37)					.145	-.033	-.156	.052
Negative Mood (NM)	13.21 (3.99)						.200	.056	.190
Systolic Pressure (SP)	112.23 (14.67)							**.688**	.061
Diastolic Pressure (DP)	73.22 (9.24)								.004
Heart Rate (HR)	77.39 (12.40)								

Participants were split into lower- and higher-level PIU groups at the cut-off for mild or worse PIU. This produced a lower-PIU group (*n* = 77; mean = 29.95 ± 5.38; range = 10–39), and a higher-PIU group (*n* = 67; mean = 51.58 ± 10.03; range = 40–73). The number of type of websites visited during the 15min internet session by each participant was retrieved from their browsing history. Five participants had deleted their browsing history, but the mean number of websites visited during the 15min period by the remaining participants was 3.17 (± 1.62, range 1–14): 2.95 (± 1.28, range 1–7) sites for lower-PIU scorers; and 3.12 (± 1.91, range 1–14) sites for the higher-users. A t-test revealed no statistically significant difference between the groups, *t*(137) = 1.72, *p* > .08, *d* = .28. The nature of the sites visited were categorised, and the percentage of these sites were highly similar across the two PIU groups: Social Network = 40% for lower-PIU scorers, and 43% for higher-PIU scorers; E-mail = 28% for lower-PIU scorers, and 20% for higher-PIU scorers; Games = 15% for lower-PIU scorers, and 19% for higher-PIU scorers; News and Sport = 10% for lower-PIU scorers, and 8% for higher-PIU scorers; Shopping and Banking = 7% for lower-PIU scorers, and 10% for higher-PIU scorers. A chi-squared test conducted on the numbers of site visited revealed no statistically significant difference between the groups, chi-square < 1.

**Fig 1:**
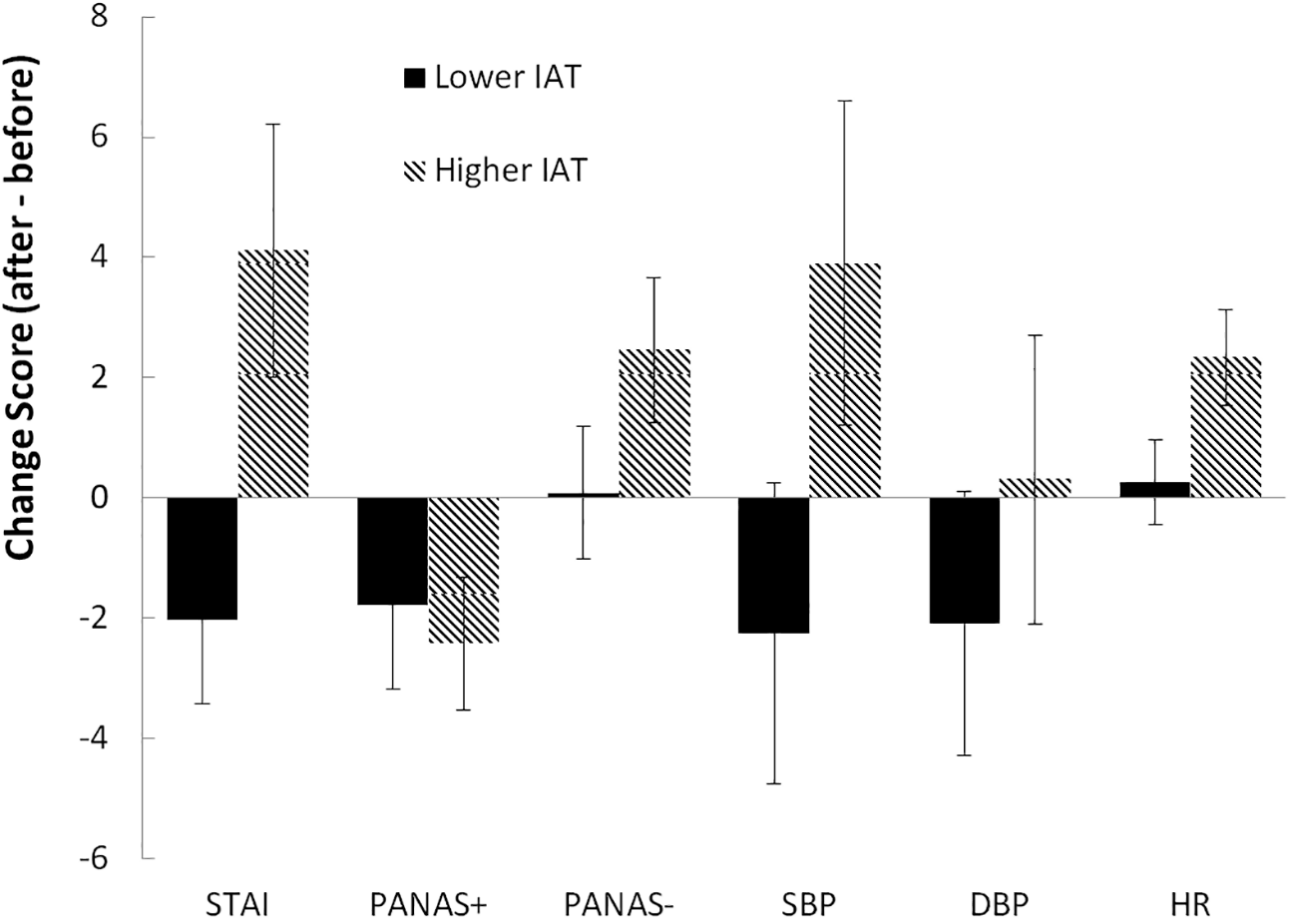
Change scores for variables (after minus before) for lower- and higher-IAT groups. STAI = state anxiety; PANAS+ = positive mood; PANAS- = negative mood; SBP = systolic blood pressure; DBP = diastolic blood pressure; HR = heart rate. Error bars = 95% confidence intervals.

Fig 1 shows the change scores (after internet cessation minus before internet session) for the lower- and higher-scoring PIU groups. The differences in the change scores between the groups (lower- versus higher-PIU scorers) were analyzed for each variable using an analysis of covariance, with depression and trait anxiety as covariates. There were significantly greater changes (after applying a Bonferroni correction: .05/6 = .008) in the higher-PIU compared to the lower-PIU group for: state anxiety, *F*(1,140) = 20.96, *p* < .001, *η*^*2*^_*p*_ = .130; negative mood, *F*(1,140) = 9.41, *p* < .003, *η*^*2*^_*p*_ = .063; systolic blood pressure, *F*(1,140) = 11.29, *p* < .001, *η*^*2*^_*p*_ = .075; and heart rate, *F*(1,140) = 12.72, *p* < .001, *η*^*2*^_*p*_ = .083, but not for positive mood, *F* < 1,*η*^*2*^_*p*_ = .005; or diastolic blood pressure, *F*(1,140) = 1.07, *p* > .30,*η*^*2*^_*p*_ = .008.

For the higher-PIU group (after applying a Bonferroni correction: .05/6 = .008), there were significant increases after the internet session in: state anxiety, *t*(66) = 3.79, *p* < .001, *d* = .46; negative mood, *t*(66) = 3.98, *p* < .001, *d* = .48; systolic blood pressure, *t*(66) = 2.81, *p* = .007, *d* = .35; and heart rate, *t*(66) = 5.40, *p* < .001, *d* = .66; a significant decrease in positive mood, *t*(66) = 4.30, *p* < .001, *d* = .52; but no change for diastolic blood pressure, *t*(66) = .26, *p* > .25, *d* = .03.

For the lower-PIU group (after applying a Bonferroni correction: .05/6 = .008), there was a significant decrease in state anxiety, *t*(76) = 2.81, *p* = .006, *d* = .32; but no change for: positive mood, *t*(76) = 2.61, *p* = .011, *d* = .28; negative mood, *t*(76) = .14, *p* > .25, *d* = .01; systolic blood pressure, *t*(76) = 1.81, *p* = .075, *d* = .20, diastolic blood pressure, *t*(76) = 1.90, *p* = .061, *d* = .21, or heart rate, *t*(76) = .59, *p* > .25, *d* = .05.

## Discussion

These findings show that the impact from cessation of an internet session on higher-PIU scorers is not restricted to psychological variables [16–19,27]. Higher-PIU scorers also show greater increased systolic blood pressure and greater increased heart rate after cessation of an internet session compared to lower-PIU scores. This finding of an impact from cessation of an internet session on physiological indices of functioning is novel, and has some implications for the manner in which such a potential internet-usage disorder is viewed. Although some physiological measures previously have been found to correlate with IAT scores [40,41], this is the first demonstration that termination of internet use produces a differential increase in such measures for higher self-reported PIU scorers. The current data also corroborate findings reported in previous studies of the psychological impact of cessation of internet sessions for those with higher self-reported PIU; that is, on cessation of internet usage, those with higher self-reported PIU show a larger increase in state anxiety and negative mood than those with lower-PIU scorers [16–19].

These cessation-of-internet effects in those with higher-PIU are similar to those noted after termination of many depressant substances; such as alcohol [21], cannabis [22], and opiate-based drugs [23]. In all of these cases, cessation of substance usage produces an increase in many measures of physiological function [21–23]. In contrast, data from stimulant-type drugs have often shown a decrease in these physiological signs; for example, nicotine [26], and MDMA [24,25]. The pattern of results from the current study, thus, suggests that those with higher-PIU scores may be experiencing withdrawal effects similar to those seen for such ‘sedative’ substances. It should be remembered that there are alterations in physiological responses associated with other types of psychiatric disorders than addictions; such as with anxiety-related problems [42,43]. However, for these disorders, physiological changes tend to be noted most pronouncedly during periods of exposure to the situations that provoke expression of the disorder, rather than being manifest on termination of the exposure.

An implication of these findings is that, for the higher-PIU scorers in this sample, the internet is possibly being used to relieve or escape stress and/or reduce anxiety [27], either produced by separation from the internet [19] or from pre-existing factors in an individual’s life [8,9]. This view derived from the physiological data is supported by consideration of the effects of internet cessation on the psychological states of the participants, in that removal of internet connection for those with higher-PIU scores increased their state anxiety and negative mood. Moreover, the psychological components of anxiety and stress have often been noted to be associated with increases in systolic, but not in diastolic, blood pressure in a variety of situations [42,43], which is the pattern of data recorded in the current study with respect to the internet. Further support for the above anxiety-escape interpretation of PIU is given by the relationship between heightened trait anxiety and higher-PIU scores found in this, and previous [8,17], explorations of the area. Individuals displaying trait anxiety may well find reduction in this state, through activities like internet use, serve to reinforce its usage [27]. There are, of course, numerous alternative possibilities as to why any addiction (substance or behavioral) is maintained at the level of the individual, including both positively and negatively reinforcement, and there may well be variations in such maintaining factors in terms of PIU [44].

One further implication of such a pattern of use concerns its potential impact on the long-term health of higher-PIU scorers [14,15,28,30]. The pattern of use of the internet in those with PIU typically involves between 7–9 hours per day [19]; overall, the participants in the current study used the internet for 5 hours per day, with higher usage in those with higher-PIU scores. These observations seem to suggest that internet use has become a normative activity, especially for younger individuals (which, of course, does not mean that it is risk-free). However, this high amount of use often tends to be distributed across numerous short visits [19,27] (modeled by the relatively short 15min use in the current study) before cessation. The constant separation, re-connection, and separation, and resultant psychological and physiological stress that this may impart [16,19], may impact a range of physiological systems [30,40,41], increasing risks of physical disease [28,30,42], as well as psychological distress [16–19]. For example, the stress associated with internet addiction has already been demonstrated to impact the sympathetic nervous system [40], and associated dopaminergic systems [41,42], which is potentially associated with compromised immune function in higher-PIU scorers [30]. The current results, especially those related to systolic blood pressure and heart rate [40,42], indicate that cessation of an internet session for higher PIU scorers is such a stressful event [19,27].

It should be noted that there are a number of limitations to the generality of the conclusions that might be reached on the basis of the present results. Firstly, the current sample was obtained from a western developed country, and results may be different in other cultures. While there is no reason to assume that ethnicity per se has any impact on the factors producing addictions, the cultural role that the internet may play in particular societies may impact on the maintenance of PIU. Secondly, these findings may be typical of those seen for general internet users, but such results may not be seen with other populations who may use the internet heavily for pursuits like gaming or pornography, both of which activities might be motivated by increased levels of arousal [45,46]. The types of website visited during the 15min internet session in the current study were broadly similar to the typical usage also claimed by the participants, and are similar to those seen in the typical population [12,47]. This possibility will require further exploration with specific samples. Thirdly, the exposure to the internet was, of necessity, somewhat artificial in the current laboratory study. This nature of the experiment was such that this could not be helped, although, as discussed above, the time period of the internet session and the types of usage seen in this period were in line with those noted as typical [19,47]. However, future studies might be conducted with the usage of portable physiological measurement devices that might measure internet usage *in situ*. Fourthly, the degree of PIU for the current sample was assessed by using the IAT. Scores on a single questionnaire should not necessarily be equated to establishing the presence of a clinical problem, and the participants identified as having ‘higher-problems’ in this study tended to have mild (but greater) issues than those identified as having ‘lower-problems’–it is unclear whether these relate to an addiction or actual functional impairment. Nevertheless, despite these caveats, the current data are the first to show clear physiological impacts of internet cessation in a group of higher PIU scorers, which suggests that PIU may have some similarities to ‘addiction-like’ problems.

The current data in relation to the proportion of individuals reporting higher levels of PIU are in line with previous estimates of the numbers of individuals who report some degree of PIU [12,17]. That there was little difference between the level of PIU in males and females is also consistent with recent reports, which show this previously-noted gender difference to be diminishing [17,27]. The correlations between the psychological variables and self-reported PIU prior to the internet session use also shows that these data were consistent with previous explorations of these measures: PIU scores (measured by the IAT) was associated with both trait and state anxiety [16,17], as well as with increased heart rate [40]. It should be noted, however, that the current findings with regard to the physiological impacts of internet cessation are independent of concomitant psychological factors, such as depression and trait anxiety, and also of the nature and number of websites visited by the participants during their session.

In summary, these results give further importance to the notion that excessive internet use for some individuals should be examined as a possible behavioral addiction, akin to gambling. That those who self-report higher levels of PIU demonstrate increased heart rate and systolic blood pressure on cessation of an internet session is suggestive of a ‘withdrawal-like’ effect, which is similar to that seen for a range of sedative-type drugs. This finding adds to the growing literature that suggests that any such PIU-disorder may have some addiction-like properties, and should be regarded as a potentially serious global problem.
